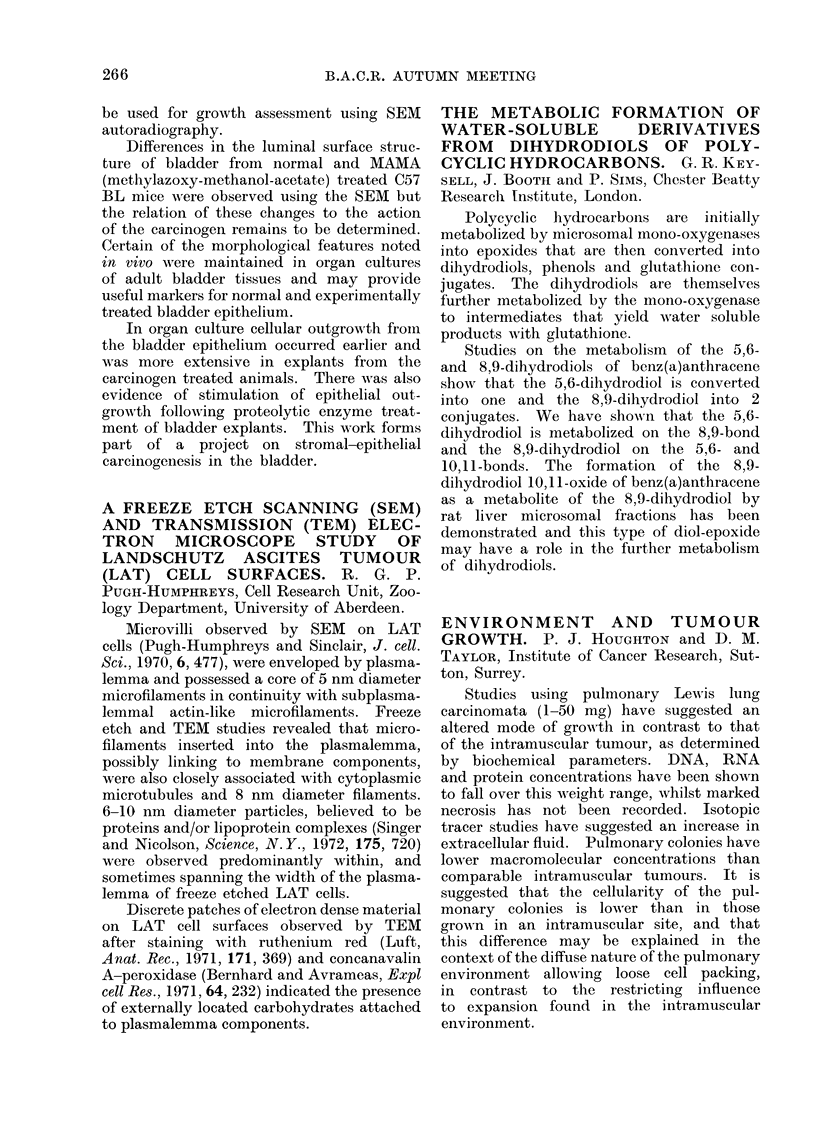# Proceedings: The metabolic formation of water-soluble derivatives from dihydrodiols of polycyclic hydrocarbons.

**DOI:** 10.1038/bjc.1975.57

**Published:** 1975-02

**Authors:** G. R. Keysell, J. Booth, P. Sims


**THE METABOLIC FORMATION OF WATER-SOLUBLE DERIVATIVES FROM DIHYDRODIOLS OF POLYCYCLIC HYDROCARBONS.** G. R. KEYSELL, J. BOOTH and P. SIMS, Chester Beatty Research Institute, London.

Polycyclic hydrocarbons are initially metabolized by microsomal mono-oxygenases into epoxides that are then converted into dihydrodiols, phenols and glutathione conjugates. The dihydrodiols are themselves further metabolized by the mono-oxygenase to intermediates that yield water soluble products with glutathione.

Studies on the metabolism of the 5,6- and 8,9-dihydrodiols of benz(a)anthracene show that the 5,6-dihydrodiol is converted into one and the 8,9-dihydrodiol into 2 conjugates. We have shown that the 5,6-dihydrodiol is metabolized on the 8,9-bond and the 8,9-dihydrodiol on the 5,6- and 10,11-bonds. The formation of the 8,9-dihydrodiol 10,11-oxide of benz(a)anthracene as a metabolite of the 8,9-dihydrodiol by rat liver microsomal fractions has been demonstrated and this type of diol-epoxide may have a role in the further metabolism of dihydrodiols.